# Novel double beta-lactam therapy for *Granulicatella adiacens* infective endocarditis

**DOI:** 10.1016/j.idcr.2021.e01082

**Published:** 2021-03-24

**Authors:** S. Khan, C. Urban, V. Singh, D. Liu, S. Segal-Maurer, Y. Parmar, J. Yoon

**Affiliations:** a The Dr. James J. Rahal, Jr. Division of Infectious Diseases, NewYork-Presbyterian Queens, 56-45 Main Street, Flushing, NY, 11355, USA; b Department of Pathology, NewYork-Presbyterian Queens, 56-45 Main Street, Flushing, NY, 11355, USA; c Department of Cardiology, NewYork-Presbyterian Queens, 56-45 Main Street, Flushing, NY, 11355, USA; d Weill Cornell Medical College, Cornell University, NY, 10065, USA

**Keywords:** Infective endocarditis, *Granulicatella adiacens*, Double beta-lactam therapy, Synergy

## Abstract

*Granulicatella adiacens*, a nutritionally variant streptococci (NVS) is a well described organism associated with endocarditis. Previously communicated cases have documented the use of double beta-lactam therapy with ampicillin and ceftriaxone to treat patients with infective endocarditis due to *Enterococcus faecalis* and *Streptocossus pneumoniae*. We describe the first case of *Granulicatella adiacens* infective endocarditis in a patient successfully treated with the combination of intravenous ampicillin and ceftriaxone and document their synergistic activity.

## Introduction

Infective endocarditis (IE) due to *Granulicatella adiacens*, a nutritionally variant streptococcus (NVS) is well described. NVS is reported to account for 5–6 % of all IE due to streptococcal species [1,2]. The current recommendation for treatment by the American Heart Association in conjunction with the Infectious Disease Society of America is ampicillin or penicillin G with gentamicin for 4–6 weeks or alternatively vancomycin [3]. This parallels the outcomes and recommendations for IE due to *Enterococcus faecalis*. Current recommendations for the treatment of IE due to *Enterococcus faecalis* include the option of double beta-lactam therapy with ampicillin and ceftriaxone [3]. We hypothesize that double beta-lactam therapy could also result in good outcomes for IE due to *Granulicatella adiacens*. Here, we report the first case of IE due to *Granulicatella adiacens* to be successfully treated with the combination of intravenous ampicillin and ceftriaxone.

## Case report

A 73 year old female with a history of four months of intermittent bright red blood per rectum and thirty pound weight loss presented to our hospital with abdominal cramping and pain. In the Emergency Department she was afebrile with a heart rate of 100 beats per minute. Initial laboratory studies revealed a hemoglobin of 6.3 g/dL and a normal white blood cell count with a normal automated differential. Colonoscopy demonstrated severe diverticulosis in the sigmoid, descending, transverse, and ascending colon.

She had a fever of 38.4 degrees centigrade following one of several transfusions of packed red blood cells. Blood cultures were drawn that yielded four out of four bottles of gram-positive cocci in chains, identified as *Granulicatella adiacens*. An infectious disease consultation was then requested. Trans-esophageal echocardiogram demonstrated a mobile, echodense lesion measuring 0.9 by 0.6 cm attached to the aortic valve with associated severe aortic regurgitation. The cardiothoracic surgery department was consulted and recommended that the patient may eventually require aortic valve replacement but recommended initially implementing conservative management with intravenous antimicrobials.

Susceptibilities by E-testing for ampicillin and ceftriaxone alone and then in combination at standard concentrations which demonstrated synergy was performed by our microbiology laboratory (Table 1). The patient required daily intravenous and oral furosemide to control her congestive heart failure and we deemed her high risk for nephrotoxicity with prolonged aminoglycoside use. After consulting with the patient and her family, we decided to treat her with double beta-lactam therapy with combination of ampicillin 2 g intravenously every 6 h and ceftriaxone 2 g intravenously every 24 h, based on her creatinine clearance and serum albumin, levels for 6 weeks. She was discharged to a nursing home to complete her treatment.

**Table 1:** Fractional inhibitory concentrations of antimicrobials alone and in combination.

Antimicrobials	Minimal Inhibitory Concentration (ug/mL) by E test		Fractional Inhibitory Concentration (ug/mL)
Ampicillin alone	actual	0.25	0.016/0.25 = 0.064
Ceftriaxone alone	actual	0.19	0.016/0.19 = 0.084
Ampicillin plus ceftriaxone	actual	0.016	0.148

Seven months later she presented to our Emergency Department with asthma exacerbation. She had no signs or symptoms of IE and her blood cultures were negative. She was then transferred to another institution for aortic valve replacement. Eleven months after treatment for her IE she had recovered well from her valve replacement (personal communication).

## Discussion

We present the first case of IE due to *Granulicatella adiacens* successfully treated with combination of intravenous ampicillin and ceftriaxone. The current guidelines for the treatment of IE due to this bacteria is intravenous ampicillin or penicillin G plus gentamicin. This recommendation is based on historically poor outcomes with ampicillin or penicillin G alone despite excellent susceptibilities. Clinical failure has been documented with high dose penicillin G alone despite in vitro susceptibility of the organism to both the inhibitory and bactericidal action of low concentrations of penicillin [4]. Rabbit model studies demonstrate that outcomes with penicillin G and an aminoglycoside were superior to penicillin G alone [5]. This parallels the outcomes and recommendations for treating IE due to *Enterococcus faecalis*. Although inherently resistant to ceftriaxone, when combined with ampicillin or penicillin G, there is broader binding to penicillin binding proteins (PBPs) leading to synergistic activity against *Enterococcus faecalis*. This has now been demonstrated in vitro, in vivo, and in animal studies [6].

NVS has gone through various taxonomic changes. They are now recognized as two distinct genera, *Abiotropia* and *Granulicatella*. Some of these distinctions were based on the identification of various PBPs. The precise function of these PBPs are not well understood and underscore the need for further research. We hypothesized that using double beta-lactam therapy for IE due to *Granulicatella adiacens*, similar to *Enterococcus faecalis*, will result in synergy, enhanced killing, and good clinical outcome without the associated risks with the use of aminoglycosides. This case highlights a potential safe and effective, alternative treatment for IE due to NVS.

## CRediT authorship contribution statement

**Shanza Khan:** Participated in the initial writing of the manuscript. **Carl Urban:** Participated in the initial writing, review and editing of the manuscript. **Vishnu Singh:** Participated in the diagnostic process. **Dakai Liu:** Participated in the diagnostic process, review and editing of the manuscript. **Sorana Segal-Maurer:** Participated in the review and editing of the manuscript. **Yuvrajsinh Parmar:** Participated in the diagnostic process. **James Yoon:** Participated in the diagnostic process, writing, review and editing of the manuscript.

## Funding

This research did not receive any specific grant from funding agencies in the public, commercial, or not-for-profit sectors.

## Patient consent

Consent to publish was not obtained since the case report does not contain any personal identifiers.

## Declaration of Competing Interest

The authors report no declarations of interest.
